# Sarcopenic obesity and skeletal development in children: recent advances

**DOI:** 10.3389/fped.2026.1836925

**Published:** 2026-07-13

**Authors:** Yumo Liu, Xinyu Li, Xuhan Liu

**Affiliations:** 1 Department of Endocrinology, Dalian Municipal Central Hospital Affiliated to Dalian University of Technology, Dalian, China; 2 Department of Graduate School, Dalian Medical University, Dalian, China

**Keywords:** body composition, bone health, bone metabolism, children, muscle–bone interaction, obesity, peak bone mass, sarcopenic obesity

## Abstract

Childhood obesity has become one of the most prevalent chronic health conditions and represents a major risk to normal growth and development in pediatric populations. As its global incidence continues to rise, increasing attention has been directed toward sarcopenic obesity (SO) as an important and emerging phenotype. This condition is defined by the coexistence of excessive adiposity and diminished muscle mass, which can negatively influence physical development as well as skeletal health in children. During childhood and adolescence, skeletal growth progresses rapidly and is highly sensitive to metabolic and hormonal influences. In this context, SO may impair bone formation via mechanical loading alterations, endocrine dysregulation, and chronic low-grade inflammation, ultimately contributing to reduced bone mineral content. Moreover, the combined impact of excess fat and insufficient muscle mass during these critical developmental stages may have long-term consequences that extend into adulthood, highlighting the importance of timely prevention and intervention. This narrative review examines the relationship between sarcopenic obesity and bone mass in children. It synthesizes recent evidence on epidemiology, underlying pathophysiological mechanisms, clinical features and evidence, as well as screening and management strategies, and further proposes recommendations focused on lifestyle-based interventions.

## 1 Introduction

According to a report from the WHO, more than 340 million children and adolescents aged 5–19 were classified as overweight or obese worldwide in 2016 (1). Pediatric obesity has therefore emerged as a major global public health concern. It is not only strongly linked to metabolic disorders—including insulin resistance, type 2 diabetes, hypertension, dyslipidemia, and MAFLD—but also exerts significant influences on the development of multiple organ systems, particularly the musculoskeletal system. The concept of sarcopenia was initially introduced by Rosenberg to describe age-related loss of muscle mass in older adults. However, accumulating evidence suggests that varying degrees of muscle deficit are also present in children and adolescents, and these changes are closely related to metabolic abnormalities such as insulin resistance and metabolic syndrome. When reduced muscle mass occurs concurrently with excess adiposity, the condition is termed sarcopenic obesity, which is characterized by increased fat accumulation alongside diminished muscle mass. Importantly, SO constitutes a synergistic state in which excess adiposity and muscle deficiency interact to aggravate skeletal fragility beyond their independent effects (13). Nevertheless, a standardized definition and diagnostic framework for pediatric SO remain lacking. Most existing studies rely on criteria derived from adult populations or apply cut-off values based on different body composition parameters, which limits both epidemiological comparability and clinical utility. Meanwhile, childhood and adolescence represent crucial periods for rapid bone accrual and the achievement of peak bone mass (PBM) (1). During these stages, there is a strong functional interaction between muscle and bone, whereby skeletal muscle enhances bone formation through mechanical loading and plays a central role in the acquisition of bone mass.

It should be emphasized that the influence of obesity and muscle loss on bone metabolism is multifaceted and operates in a bidirectional manner. Increased body weight may enhance bone mass to a certain degree through greater mechanical loading; however, obesity-related factors—such as chronic low-grade inflammation, insulin resistance, and endocrine disturbances—can suppress bone formation while accelerating bone resorption. At the same time, diminished muscle mass reduces mechanical stimulation of the skeleton, thereby impairing its adaptive response (2). In this context, the coexistence of excess adiposity and insufficient muscle mass further complicates their overall effects on skeletal health, with evidence suggesting potential synergistic interactions that intensify their impact (3). Clinical findings indicate that children with obesity frequently present with impaired bone development and decreased bone density, and these alterations may extend into adult life, increasing susceptibility to osteoporosis and fractures. This concern is particularly critical during the period of PBM acquisition, as inadequate bone accumulation at this stage can have long-term consequences for skeletal strength. Accordingly, there is a need for comprehensive investigation into the epidemiological patterns, underlying mechanisms related to bone metabolism, and clinical features of pediatric SO. In addition, summarizing effective approaches for screening and intervention is essential to support early detection and management, ultimately providing a solid theoretical foundation for clinical practice (10, 11, 15).

## 2 Epidemiology of sarcopenic obesity in children

### 2.1 Definition and diagnostic challenges of sarcopenic obesity in children

As SO gains recognition as a clinically meaningful and distinct phenotype, the absence of standardized definitions and diagnostic criteria has become a major challenge in pediatric research. In principle, pediatric SO may be described as the simultaneous presence of excess adiposity and relatively reduced muscle mass and/or function, with consideration given to variables such as age, sex, and stage of development. At present, no universally accepted international criteria exist for diagnosing SO in children, which represents a fundamental limitation for both epidemiological investigations and clinical practice. In contrast to adults, pediatric populations undergo continuous growth and maturation, accompanied by substantial physiological fluctuations in both muscle and fat compartments, thereby increasing the complexity of defining this condition (4, 15).

Current research generally adopts operational definitions of SO based on body composition parameters. Muscle mass is most often evaluated using dual-energy x-ray absorptiometry (DXA), bioelectrical impedance analysis (BIA), or imaging techniques such as MRI and CT. Commonly applied indices include appendicular skeletal muscle mass adjusted for height squared (ASM/height^2^) and ASM relative to BMI. Nevertheless, the normative ranges for these metrics are frequently extrapolated from adult cohorts or heterogeneous populations, and standardized pediatric-specific cut-off values remain lacking (4). Beyond quantitative assessment of muscle mass, functional measures—such as handgrip strength—are increasingly incorporated into diagnostic frameworks. Evidence suggests that relative grip strength may serve as a practical indicator for identifying children at risk of SO; however, universally accepted thresholds have not yet been defined. In addition, variability in measurement consistency and differences in accessibility across assessment modalities further contribute to diagnostic ambiguity (37, 38). Importantly, these methodological discrepancies directly result in considerable heterogeneity in reported prevalence rates among studies, thereby complicating cross-population comparisons and hindering the effective translation of research evidence into clinical application.

Importantly, direct application of adult SO definitions to children may lead to misclassification. Unlike adults, children experience continuous changes in muscle mass, fat distribution, muscle strength, and physical performance throughout growth and puberty (1, 4). Therefore, diagnostic thresholds established in adult populations may not accurately reflect pathological alterations in pediatric body composition. Future consensus definitions should incorporate age-, sex-, and pubertal stage-specific reference values. In summary, the diagnosis of SO in pediatric populations continues to depend largely on research-oriented definitions, with a lack of consistent and widely applicable criteria. Developing a standardized diagnostic framework is therefore imperative. Such a system should be stratified according to age, sex, and stage of development, while integrating both quantitative assessments of muscle mass and functional performance measures, so as to enhance diagnostic precision and clinical applicability (Table 1).

**Table 1:** Components and assessment methods used for the diagnosis of pediatric sarcopenic obesity.

Domain	Common indicators	Assessment methods	Current limitations
Muscle mass	ASM/height^2^, ASM/BMI, SMI	DXA, BIA, MRI, CT	No universally accepted pediatric cut-offs
Muscle strength	Handgrip strength, Relative grip strength	Dynamometer	Limited reference values across age groups
Physical function	TUG, 6MWT, gait speed	Functional performance tests	Rarely incorporated into pediatric definitions
Adiposity	BMI, fat mass percentage, waist circumference	Anthropometry, DXA, BIA	Variable obesity definitions

### 2.2 Epidemiology

The *2024 World Obesity Report* highlights a continued global increase in the prevalence of overweight and obesity (5). The number of affected adults is expected to grow from 2.2 billion in 2020 to 3.3 billion by 2035, with prevalence rising from 42% to 54%. Concurrently, among individuals aged 5–19 years, the affected population is projected to expand from 430 million to 770 million, with prevalence increasing from 22% to 39%. These estimates suggest that by 2035, roughly two in five adolescents worldwide may be classified as overweight or obese. Within this context, pediatric SO is receiving increasing attention as a clinically relevant phenotype. Nevertheless, available epidemiological evidence remains highly variable. Reported prevalence rates differ markedly across studies, ranging from 5.66% to 69.7% in girls and from 7.2% to 81.3% in boys, largely reflecting inconsistencies in diagnostic criteria, measurement approaches, and population characteristics (1). These findings are supported by multiple primary studies conducted in school-aged children and adolescents (46, 47). Among children who are already overweight or obese, the proportion identified with SO tends to be higher, with some studies indicating rates between 10% and 40%, pointing to a substantial burden within this subgroup. Furthermore, in regions undergoing rapid economic growth and lifestyle Westernization, the risk of pediatric SO may rise further due to increased consumption of energy-dense diets and declining levels of physical activity. At present, cross-national comparisons of prevalence are hindered by the absence of standardized criteria, resulting in limited comparability of existing data (47). Therefore, large-scale studies employing unified methodologies are needed to validate and refine current epidemiological estimates (Table 2).

**Table 2:** Reported prevalence of pediatric sarcopenic obesity.

Study	Population	Sex	Age range	Prevalence
Zembura et al. (2022) (1)	General pediatric populations	Girls	Various	5.66%–69.7%
Zembura et al. (2022) (1)	General pediatric populations	Boys	Various	7.2%–81.3%
Multiple studies	Overweight/obese children	Mixed	Various	10%–40%

Available studies frequently stratify results by age and sex; however, relatively few studies report outcomes according to pubertal stage, limiting direct comparisons.

### 2.3 Critical appraisal of current evidence

While accumulating studies provide valuable insights into pediatric SO and bone health, several limitations must be acknowledged. Most studies are cross-sectional, limiting the ability to draw causal inferences. Substantial heterogeneity exists in diagnostic definitions, measurement modalities, and study populations. Risk of bias arises from small sample sizes, inconsistent assessment methods, and potential publication bias. Moreover, confounding factors such as pubertal stage, sex hormones, and ethnicity are not consistently controlled, which may influence observed associations. Reverse causality cannot be excluded, as reduced bone mass may also affect muscle development. Overall, the current evidence provides preliminary associations but underscores the need for longitudinal, standardized studies to strengthen causal inference and inform clinical practice.

## 3 Clinical effects of sarcopenic obesity on bone mass

### 3.1 Adolescence: the golden window for peak bone mass accumulation

Adolescence represents a pivotal phase of accelerated growth in children, during which marked alterations in body composition occur. Evidence suggests that boys typically show a progressive decline in body fat percentage alongside a substantial increase in fat-free mass (FFM), whereas girls tend to accumulate more fat mass and demonstrate comparatively slower gains in muscle. These sex-specific patterns are largely driven by hormonal changes during puberty (6). Such dynamic shifts in body composition render adolescence a particularly vulnerable period for the development of SO. During this stage, excessive fat accumulation may coincide with relative deficits in muscle mass, thereby facilitating the emergence of metabolic disturbances, including insulin resistance and metabolic syndrome. Concurrently, this developmental window is also critical for the attainment of PBM. Evidence indicates that a substantial proportion of total lifetime bone mass is accrued during childhood and adolescence, with a significant fraction occurring during the adolescent growth spurt; by approximately 18 years of age, around 90% of PBM has been achieved (7, 54). Notably, bone mass may increase by more than one-quarter within the period spanning roughly two years before and after peak height velocity. In addition to rapid gains in bone mineral density (BMD), important changes also occur in bone microarchitecture—such as trabecular number and thickness—as well as in overall bone geometry. Supporting these observations, a longitudinal study of pubertal boys demonstrated that leptin levels and bone turnover markers were significantly associated with changes in BMD and bone mineral content (BMC) throughout puberty, highlighting the importance of metabolic and hormonal regulation during skeletal development (51). The level of PBM achieved has important implications for long-term skeletal health. It has been reported that a 10% higher PBM is associated with an estimated 50% lower risk of fractures later in life. Therefore, adolescence constitutes not only a key window for bone mass acquisition but also a decisive stage influencing lifelong skeletal strength (1, 7). Sex-specific differences in body composition and skeletal development should be considered when assessing pediatric sarcopenic obesity. Boys and girls exhibit distinct patterns of fat accumulation, muscle growth, and bone accrual during adolescence, influenced by hormonal changes (8, 9). These differences may modulate both the prevalence and clinical consequences of sarcopenic obesity, underscoring the importance of sex-stratified analyses in future research.

### 3.2 Asynchrony in musculoskeletal development during the rapid growth phase

During adolescence, the rapid growth of the musculoskeletal system does not occur in a fully coordinated manner between bone and muscle. Evidence from imaging analyses and longitudinal cohort studies indicates that increases in bone length generally precede gains in muscle volume. For instance, the peak velocity of tibial elongation is often observed approximately 1–2 years before the maximum rate of lower-limb muscle growth (8). This pattern is evident in both males and females, suggesting that a degree of “muscle–bone asynchrony” is an inherent feature of normal development. In periods of accelerated linear growth, bones undergo rapid elongation, whereas muscles may not yet have achieved corresponding increases in size and functional capacity. This mismatch can lead to relatively reduced mechanical support and traction exerted by muscle on bone. Some researchers have proposed that such temporal discrepancies among bone, muscle, and ligament development may transiently render the growth plate and surrounding bone tissue more vulnerable, thereby elevating the likelihood of sports-related injuries and fractures (9). From a metabolic standpoint, bone mineral accrual is a dynamic, non-linear process that relies heavily on mechanical stimuli generated by muscle activity. Repeated mechanical loading promotes bone formation and enhances cortical structure; conversely, delayed muscle development results in diminished mechanical stimulation, which may impair osteogenesis and limit bone mass accumulation. Consistent evidence demonstrates a positive association between muscle mass and both bone density and strength. In contrast, excessive adiposity—particularly visceral fat—can negatively influence bone remodeling through the release of pro-inflammatory cytokines, such as TNF-α and IL-6, which suppress bone formation and enhance resorption (10, 11). Taken together, during the adolescent growth spurt, a condition in which bone elongation exceeds the pace of muscle adaptation may not only heighten the short-term risk of injury and fractures but also compromise the efficiency of bone mass acquisition, thereby affecting the achievement of PBM. Moreover, when combined with increased fat accumulation and reduced muscle mass, as seen in SO, this imbalance within the musculoskeletal system may be further intensified, leading to sustained detrimental effects on bone quality.

### 3.3 Clinical evidence regarding the impact on bone mass

At present, evidence directly examining the impact of SO on bone mass in pediatric populations remains relatively scarce; nevertheless, available studies offer valuable insights from several angles. From the perspective of bone phenotype, children with obesity often demonstrate greater BMC and BMD than their normal-weight peers. However, once these indices are normalized for body weight or body surface area, this apparent advantage diminishes and may even reverse (12). This pattern of “absolute increase but relative deficit” suggests that although excess body weight imposes greater mechanical loading on the skeleton, bone strength may not be proportionally adapted to support it.Consistent with these observations, a comprehensive review has shown that, compared with children of normal weight, those with obesity have at least a 25% higher likelihood of peripheral fractures. In cases of severe obesity, the risk of fractures involving the lower extremities—such as the ankle, knee, and long bones—is even more pronounced (3). A longitudinal cohort study of 466,997 children aged 4–15 years showed that preschool obesity was associated with a significantly higher incidence of childhood fractures compared with normal—weight peers. This finding has also been confirmed by several basic studies conducted in community and clinic-based cohorts (48–50). These outcomes are not solely attributable to greater impact forces related to body mass but are also closely linked to reduced muscle strength and impaired motor coordination, both of which increase susceptibility to falls. In this context, the concept of “osteosarcopenic obesity” has been introduced to describe a composite condition characterized by excess adiposity, diminished muscle mass, and inadequate bone mass. Individuals with this phenotype face a substantially higher risk of fractures and functional decline compared with those presenting with obesity or sarcopenia alone, providing an important framework for understanding the cumulative skeletal burden associated with SO (2, 13). Further support is derived from studies stratified by body composition. After adjusting for confounding variables such as height and weight, children with SO have been shown to exhibit lower BMC and BMD than obese peers without SO (2). A recent prospective study similarly reported that, among overweight or obese children, those with reduced muscle mass displayed significantly lower whole-body (excluding skull) BMC and BMD compared with non-sarcopenic counterparts (14). Additionally, a significant positive relationship between grip strength and whole-body bone density was observed, indicating that declines in muscle function and bone mass may occur simultaneously. From a bone metabolism standpoint, Available evidence suggests that SO is not only associated with metabolic disturbances but may also elevate the risk of adverse skeletal outcomes, including osteoporosis, although much of this evidence is derived from adult populations (1). In obese adolescents, markers of bone formation, such as osteocalcin, have been found to correlate positively with muscle mass, whereas indicators of bone resorption tend to be increased in those with sarcopenic obesity, further reflecting an imbalance in bone remodeling processes. Although long-term pediatric data remain limited, findings from adult studies provide supportive indirect evidence: obese adults with SO exhibit lower bone mineral content (e.g., mean 2.56 kg) compared with those without SO (2.85 kg), along with a higher prevalence of reduced bone mass or osteoporosis (2). Overall, available evidence suggests that SO exerts more detrimental effects on skeletal health than obesity alone—including bone phenotype, fracture susceptibility, and body composition analyses—that SO exerts more detrimental effects on skeletal health than obesity alone. The combined state of increased fat mass and reduced muscle mass may impair bone accrual and disrupt normal remodeling. During adolescence, a crucial period for achieving PBM, the presence of SO may have long-lasting consequences for skeletal integrity, underscoring the need for heightened clinical awareness.

In addition to sarcopenic obesity (SO), osteosarcopenic obesity (OSO) has been proposed as a broader concept describing combined muscle, bone, and adipose impairment. OSO provides a unifying framework to conceptualize the synergistic interaction between excess adiposity, reduced muscle mass, and compromised bone health. This integrated phenotype highlights how adipose tissue, skeletal muscle, and bone collectively contribute to skeletal fragility beyond the independent effects of obesity or sarcopenia alone. Recognizing OSO in pediatric populations can facilitate early risk stratification and support comprehensive lifestyle-based interventions targeting both muscle and bone while controlling adiposity (Table 3).

**Table 3:** Summary of clinical studies evaluating sarcopenic obesity and skeletal outcomes in children.

Study	Population	Age/pubertal status	SO definition	Main bone outcome	Key findings
Czeck et al. (2023) (12)	Children and adolescents with obesity	Adolescents	Body composition-based	BMC/BMD	Bone indices increased in absolute terms but reduced after adjustment for body size
Fintini et al. (2020) (3)	Pediatric obesity	Mixed ages	Obesity-related skeletal studies	Fracture risk	≥25% higher fracture risk in obese children
Mikulska et al. (2026) (14)	Overweight/obese children	School-age children	Reduced muscle mass	BMC/BMD	Lower whole-body BMC and BMD in sarcopenic group
De Lorenzo et al. (2023) (2)	SO populations	Mixed	Osteosarcopenic obesity framework	Bone mass	Lower BMC and BMD compared with obesity alone

Available studies frequently stratify results by age and sex; however, relatively few studies report outcomes according to pubertal stage, limiting direct comparisons.

## 4 Mechanisms of action of sarcopenic obesity on bone metabolism

Bone development involves both linear growth and bone mass accumulation, and childhood and adolescence represent a critical window for skeletal growth and development. During this period, bone formation predominates, and bone modeling and bone turnover are highly active, playing a decisive role in shaping bone volume and morphology and ensuring the steady accumulation of bone mass. The acquisition of bone mass depends largely on mechanical loading exerted by muscles and associated biological signals. Furthermore, bone development in children is regulated by a combination of factors, including genetics, endocrinology, nutrition, physical activity, and disease (15, 16). The effects of SO on bone metabolism are not driven by a single factor but result from the combined action of multiple pathways. Overall, the impact of SO on the skeleton can be understood from multiple perspectives, including endocrine regulation, mechanical loading, and inflammation/adipokines.

### 4.1 Endocrine regulation

Endocrine regulation plays a pivotal role in bone metabolism disturbances associated with SO, with the growth hormone–insulin-like growth factor 1 (GH–IGF-1) axis and sex hormones constituting a central regulatory network. In individuals with obesity, persistent hyperinsulinemia can inhibit growth hormone secretion and attenuate its pulsatile release, thereby disrupting the physiological activity of the GH–IGF-1 axis. Although circulating IGF-1 concentrations in some obese children may remain within normal limits, functional impairment of this axis can still contribute to metabolic alterations affecting both cortical and trabecular bone (17). IGF-1 is a critical mediator of bone formation, as it promotes chondrocyte proliferation and osteoblast differentiation. Consequently, dysfunction of the GH–IGF-1 axis may indirectly reduce bone-forming capacity. In addition, skeletal muscle functions as an endocrine organ by releasing myokines, such as irisin and myostatin, which participate in the regulation of bone metabolism. Together with anabolic factors including IGF-1, these signaling molecules contribute to muscle–bone crosstalk. Under conditions of reduced muscle mass, this beneficial signaling is diminished, further limiting bone mass accrual. Sex steroids also exert essential effects on skeletal development during puberty and interact closely with the GH–IGF-1 axis. In the context of obesity, enhanced aromatase activity in adipose tissue facilitates the conversion of androgens into estrogens, resulting in relatively increased estrogen levels and reduced concentrations of free testosterone. Estrogen has a biphasic, dose-dependent influence on bone metabolism. During early puberty, lower estrogen levels stimulate GH secretion and enhance IGF-1 activity, promoting growth plate chondrocyte proliferation and linear growth. In contrast, in later stages of adolescence, higher estrogen concentrations accelerate epiphyseal closure by inducing chondrocyte differentiation and apoptosis, thereby restricting final adult height (18). As a result, obese children often exhibit suppressed GH secretion alongside earlier activation of sex hormones, which may lead to “developmental asynchrony,” characterized by advanced bone age but suboptimal bone mass accumulation. This mechanism represents an important endocrine pathway through which obesity influences skeletal health (55, 56).

Extending from the role of endocrine regulation, nutritional factors further influence bone remodeling by shaping metabolic status and act synergistically in the context of SO. Children affected by SO frequently demonstrate suboptimal dietary habits, characterized by excessive caloric intake alongside inadequate consumption of high-quality protein, calcium, and vitamin D. This imbalance—marked by excess energy yet insufficient essential nutrients—can directly impair bone mineralization (15). Among these factors, vitamin D deficiency is particularly prevalent in obese pediatric populations and is closely linked to disturbances in bone metabolism. Increased adiposity promotes the sequestration of vitamin D within lipid-rich tissues, thereby reducing its bioavailability. At the same time, metabolic demands and utilization of vitamin D are elevated, contributing to decreased circulating levels of 25-hydroxyvitamin D (19, 35). Insufficient vitamin D reduces intestinal calcium absorption, leading to compensatory elevations in parathyroid hormone (PTH), which subsequently enhances bone resorption and suppresses bone formation. In addition, deficiencies in other micronutrients, such as magnesium and vitamin K, may further impair bone quality by disrupting mineralization processes and the function of bone-related proteins. Overall, endocrine disturbances associated with obesity interact with chronic low-grade inflammation, shifting bone metabolism from a state favoring formation toward one dominated by resorption, ultimately resulting in decreased bone density. Importantly, current evidence indicates that children with SO experience more pronounced reductions in bone density and quality compared with those who have obesity alone (2). Experimental studies provide further mechanistic insights: for example, fat-deficient mouse models demonstrate increased trabecular bone mass and improved mechanical strength, underscoring the regulatory role of adipose tissue in skeletal metabolism (20). Although vitamin D is essential for bone health, clinical findings regarding the effectiveness of supplementation in improving bone mass in children remain inconsistent, suggesting that outcomes may vary depending on baseline nutritional status and individual metabolic characteristics (21).

### 4.2 Mechanical loading and muscle–bone interaction

Skeletal muscle contractions generate mechanical forces that are transmitted to bone during movement, thereby providing mechanical loading that plays a fundamental role in stimulating bone formation and maintaining skeletal strength (57). Under physiological conditions, the dynamic loading generated by muscle contraction enhances osteoblast activity, facilitates mineral deposition, and contributes to optimal bone architecture. In the setting of sarcopenic obesity (SO), reductions in both muscle mass and strength may contribute to lower mechanical loading indirectly, as affected individuals often engage in less physical activity. This reduced activity can limit osteogenic stimulation and potentially impair bone formation. Obesity-associated loading may exert partially protective skeletal effects, but these benefits may be offset by inflammation and muscle deficiency. Although increased body weight in obesity may impose a degree of load on the skeleton, this form of “passive” mechanical input is largely derived from static gravitational forces. Compared with the dynamic stresses produced by active muscle contraction, such loading is less effective in triggering adaptive bone remodeling. Consequently, within a body composition characterized by excess adiposity and diminished muscle mass, the presumed protective influence of higher body weight on bone integrity is attenuated and may even become unfavorable (2). In addition, children with SO often display lower levels of physical activity and reduced motor performance, further compromising the skeleton's ability to respond adaptively to mechanical stimuli. Clinical and imaging evidence suggests that, relative to individuals with obesity alone, those with SO exhibit reduced bone mineral content and decreased bone strength, highlighting the critical role of muscle deficiency in the development of skeletal fragility (22, 23). Observations during growth further reinforce this relationship, as gains in muscle mass throughout adolescence show a strong positive association with bone mineral accrual, underscoring the importance of muscle-derived mechanical loading in achieving PBM (41, 42). Moreover, patterns of fat distribution also modulate skeletal metabolism, with region-specific effects on bone outcomes. For instance, accumulation of central (trunk) adiposity has been negatively associated with bone density, a relationship that appears particularly evident among adolescent males (58).

Although several biological pathways have been proposed to explain the adverse skeletal effects of SO, current evidence remains heterogeneous and context dependent. For example, obesity-related mechanical loading may exert partially protective effects on bone mass by increasing skeletal loading, particularly in weight-bearing sites. Likewise, leptin has been reported to exhibit dual actions on bone metabolism, with both osteogenic and anti-osteogenic effects depending on peripheral vs. central signaling pathways. Furthermore, the influence of Wnt/*β*-catenin signaling may vary according to developmental stage, fat distribution, and local metabolic conditions. These observations suggest that the relationship between adiposity, muscle, and bone is highly complex and may be modified by age, pubertal status, physical activity level, and body fat distribution. Therefore, mechanistic findings should be interpreted cautiously, particularly when extrapolating evidence from adult populations to children (10, 11, 24, 27).

### 4.3 The role of inflammation and adipokines

A persistent state of low-grade inflammation is a defining feature of obesity. In the context of SO, excessive adipose tissue—including fat within the bone marrow—releases a range of pro-inflammatory cytokines, such as TNF-α and IL-6, as well as adipokines like leptin and adiponectin. These mediators stimulate osteoclast differentiation through activation of the RANKL signaling pathway, while concurrently suppressing osteoblast activity. As a result, the balance between bone formation and resorption is disrupted, ultimately contributing to bone loss (24). In addition to its direct effects on bone, this inflammatory milieu also impairs muscle protein synthesis, thereby aggravating muscle depletion and establishing a detrimental cycle linking inflammation, sarcopenia, and skeletal deterioration. Bone marrow adiposity represents a key interface between fat accumulation and bone metabolism. Evidence indicates that increased marrow fat content is inversely associated with bone density and may further compromise bone strength by modifying the local microenvironment and altering the expression of functional proteins (25, 26). Accordingly, under conditions of SO, the influence of adipose tissue on skeletal health extends beyond mechanical factors and involves complex biological interactions within the bone marrow niche. At the cellular level, disrupted differentiation of bone marrow mesenchymal stem cells (MSCs) is considered a central mechanism, reflecting a shift in lineage commitment that favors adipogenesis over osteogenesis. The Wnt/*β*-catenin signaling cascade is essential for regulating the differentiation trajectory of mesenchymal stem cells (MSCs) (58, 59). When this pathway is activated, it favors osteogenic differentiation while suppressing adipocyte formation; in contrast, its inhibition redirects MSCs toward an adipogenic fate. Under conditions such as obesity or systemic inflammation associated with SO, factors derived from adipose tissue can increase the expression of Wnt antagonists, including sclerostin and DKK1. This leads to suppression of *β*-catenin signaling, enhancement of adipogenesis, and inhibition of bone formation (27, 28, 53). The evidence remains incomplete in pediatric populations. In this regulatory network, secretory products from both muscle and adipose tissue play key roles. Irisin, a myokine, supports osteoblast differentiation and limits fat cell formation by stimulating the Wnt/*β*-catenin pathway, thereby contributing to the maintenance of bone–fat homeostasis (29, 30). In contrast, myostatin—an inhibitor of muscle growth—suppresses Wnt signaling through activation of the Smad2/3 pathway and reduces the expression of osteogenic factors, ultimately hindering bone formation (31, 32). Thus, in the setting of SO, a disrupted balance of muscle-derived factors, characterized by reduced irisin and elevated myostatin, may further impair osteogenesis. In addition, adipokines contribute to the regulation of bone metabolism through bidirectional effects, further complicating the interactions among adipose tissue, muscle, and bone.

However, the effects of leptin on bone metabolism appear to be complex and context-dependent. While elevated leptin concentrations in obesity may promote RANKL expression and enhance bone resorption, leptin has also been reported to exert osteogenic effects through peripheral pathways (33, 52). In contrast, central nervous system–mediated leptin signaling may negatively regulate bone formation (34). Therefore, the net skeletal effects of leptin likely depend on age, metabolic status, adiposity distribution, and the relative contribution of peripheral vs. central signaling pathways.

**Figure 1:**
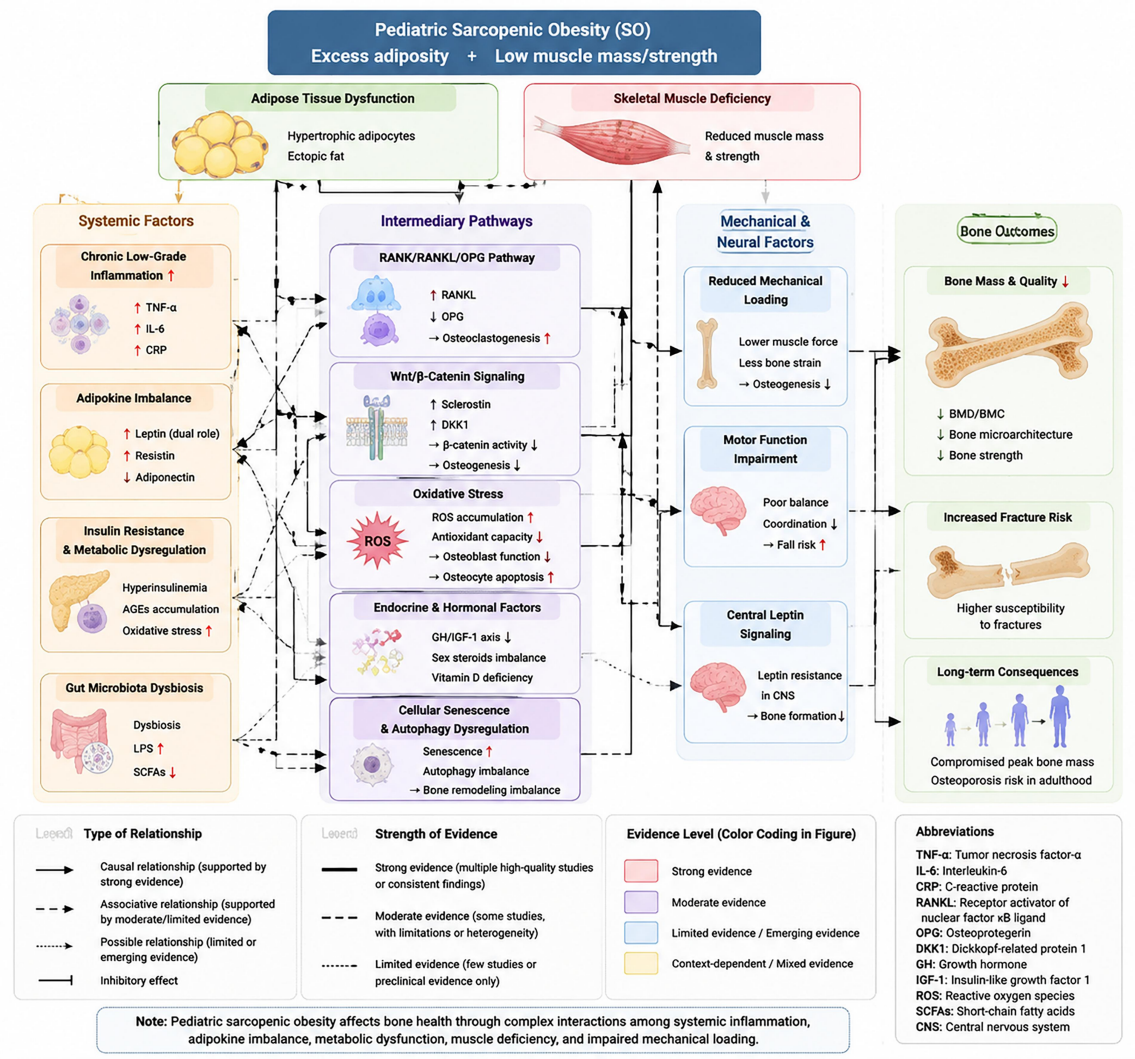
Mechanisms linking SO and bone outcomes in children. Solid arrows indicate strong evidence or likely causal relationships; dashed arrows indicate limited evidence or associative relationships.

By contrast, adiponectin generally supports bone formation and inhibits osteoclast function. Nevertheless, reduced circulating levels of this adipokine in obese individuals further compromise osteogenic capacity (35). It is also important to recognize the close interaction between inflammatory processes and endocrine dysfunction. Children with obesity frequently present with insulin resistance and compensatory hyperinsulinemia. Although insulin and IGF-1 are inherently anabolic for bone, their osteogenic effects are diminished in the presence of insulin resistance, resulting in decreased efficiency of bone formation (36). Overall, SO contributes to an imbalance in bone remodeling through multiple interacting pathways, including heightened inflammatory activity, altered adipokine signaling, and shifts in mesenchymal stem cell differentiation. These combined effects promote bone resorption while suppressing bone formation, ultimately accelerating bone loss within the skeletal microenvironment. These observations highlight the importance of muscle–bone crosstalk mediated by myokines and osteokines in the pathophysiology of pediatric sarcopenic obesity (Figure 1).

## 5 Screening and management recommendations

In light of the potential negative impact of SO on pediatric bone health, it is essential to implement screening and management approaches that emphasize early detection and risk-based evaluation. At the initial stage, all children who are overweight or obese should receive a baseline assessment that includes anthropometric measurements—such as height, body weight, body mass index (BMI), and waist circumference—along with evaluation of muscle strength, for example through grip strength or lower-limb strength testing. In addition, clinicians should obtain a history of bone pain and prior fractures to identify early signs of skeletal vulnerability. Relative grip strength (grip strength normalized to BMI) has been proposed as a practical and informative screening tool, with evidence supporting its value in identifying children at risk of SO (37). Moreover, functional performance assessments—such as the 6 min walk test or the Timed Up and Go (TUG) test—should be incorporated, as reductions in muscle strength do not necessarily parallel changes in muscle mass (38). During more detailed evaluation, body composition techniques, including dual-energy x-ray absorptiometry and bioelectrical impedance analysis, can provide simultaneous measurements of lean mass, fat mass, and bone mineral content, thereby enhancing diagnostic accuracy for SO. For individuals considered at elevated risk—such as those with impaired glucose regulation, severe obesity, or a family history of osteoporosis—additional investigations, including BMD testing or advanced imaging (e.g., CT or high-resolution bone CT), are advisable (3). At the same time, a thorough assessment of endocrine and nutritional status is warranted. This should include evaluation of vitamin D levels, markers of calcium and phosphorus metabolism, sex hormone profiles, and indicators of glucose and lipid metabolism. Given that both obesity and muscle deficiency are associated with reduced vitamin D status, periodic monitoring of serum 25(OH)D concentrations and timely corrective measures are recommended (39). For pediatric patients diagnosed with SO accompanied by decreased bone mass, early and comprehensive intervention is crucial. Management strategies should involve tailored nutritional plans aimed at optimizing intake of protein, calcium, and vitamin D, alongside resistance and weight-bearing exercise programs and behavioral modifications. These combined approaches are intended to enhance muscle mass, support bone accrual, and improve overall skeletal health.

## 6 Treatment and intervention recommendations

Given the dual adverse effects of SO in children on bone health, interventions should adopt a multidimensional, comprehensive strategy focused on nutritional optimization, physical activity promotion, and psychological and family support, with the aim of achieving synergistic improvements in muscle, fat, and bone metabolism. Current intervention recommendations for pediatric SO are supported by varying levels of evidence. While exercise interventions have been evaluated in randomized controlled trials (RCTs) and systematic reviews demonstrating beneficial effects on muscle strength and bone outcomes, evidence for specific nutritional interventions is derived primarily from observational studies, clinical guidelines, and extrapolation from broader pediatric nutrition research. Therefore, recommendations should be interpreted within the context of the available evidence base (40, 60).

### 6.1 Nutritional intervention

Adequate intake of high-quality protein is essential for promoting muscle synthesis and bone matrix formation. Current pediatric nutrition guidelines recommend a protein intake of approximately 0.85–1.2 g/kg/day for children (40). However, evidence supporting higher protein intake specifically for pediatric SO remains limited, and recommendations are largely extrapolated from general pediatric nutrition studies and observational evidence. High-quality protein sources (such as lean meat, fish, eggs, dairy products, and soy products) help improve muscle mass and promote bone formation through the muscle-bone coupling mechanism. Regarding overall dietary patterns, intake of high-sugar beverages, refined carbohydrates, and processed foods should be limited, while balanced dietary patterns (such as the Mediterranean diet) should be promoted to balance body fat control with bone nutrient supply. Increasing consumption of foods rich in calcium, vitamin D, and vitamin K (dairy products, dark green vegetables, nuts, legumes, and fish) is crucial for bone mineralization. Vitamin D plays a key role in calcium absorption and bone metabolism; vitamin D deficiency is common among obese children. It is recommended to maintain serum 25(OH)D levels above 20–30 ng/mL through moderate sun exposure and supplementation when necessary. At the same time, attention should be paid to the intake of trace elements such as magnesium and zinc, as well as antioxidant nutrients (e.g., vitamins C and E, and polyphenols), which play a supportive role in bone formation and anti-inflammatory processes. It is important to emphasize that dietary interventions should achieve energy balance while ensuring adequate nutrition, avoiding the adverse effects of excessive calorie restriction on skeletal growth (19, 21, 35).

### 6.2 Exercise intervention

Physical activity represents a fundamental strategy for enhancing both muscle mass and bone density, with resistance-based exercise playing a particularly critical role. The forces generated during muscle contraction impose mechanical stress on bone tissue, which directly activates osteoblasts and promotes mineral deposition. For optimal outcomes, resistance exercise should be integrated with weight-bearing aerobic activities (41, 42). Programs involving strength training—such as squats, push-ups, resistance band workouts, or dumbbell exercises—should begin at moderate intensity (around 50%–70% of maximal strength) and progressively increase to higher levels (70%–85%). Training is generally recommended 3–5 times per week, with sessions lasting 30–60 min and targeting major muscle groups. In parallel, weight-bearing aerobic activities, including jumping, running, and various ball sports, can further stimulate bone adaptation through repetitive impact loading (43, 44). Evidence suggests that consistent participation in resistance exercise significantly enhances bone strength in adolescents, without elevating the risk of skeletal injury when conducted with appropriate supervision. Consequently, exercise prescriptions should be individualized and guided by professionals, with particular attention to enjoyment and long-term adherence to maximize effectiveness (Table 4) (45).

**Table 4:** Evidence-based lifestyle interventions for pediatric sarcopenic obesity.

Intervention	Evidence level	Recommendation
Resistance training	RCTs + systematic reviews	3–5 sessions/week, 30–60 min/session, 50%–85% maximal strength
Weight-bearing exercise	RCTs + systematic reviews	Running, jumping, ball sports
Protein intake	Pediatric guidelines + observational studies	0.85–1.2 g/kg/day
Vitamin D optimization	Observational studies + clinical guidelines	Maintain serum 25(OH)D > 20–30 ng/mL
Family-based behavioral intervention	Systematic reviews	Improve adherence and lifestyle modification

### 6.3 Psychological and family support

Childhood obesity and SO are frequently associated with psychological distress and behavioral challenges; therefore, effective management should extend beyond physical factors to include psychosocial and family components. Interventions that adopt a family-centered approach have been shown to yield better outcomes, with active parental participation playing a pivotal role in facilitating behavioral modification. Evidence indicates that strategies involving family members are more successful than those directed solely at the child (45). Practical approaches include helping families develop balanced dietary habits and regular physical activity routines, reinforcing positive behaviors, and minimizing negative or critical feedback. For children experiencing body image dissatisfaction or reduced self-esteem, psychological support may be beneficial in promoting healthier behavioral patterns and improving long-term adherence. Furthermore, engaging families in shared health-related activities—such as joint exercise or preparing meals together—can enhance the sustainability of these interventions.

In summary, the management of pediatric SO should primarily focus on lifestyle modification. By integrating nutritional improvements, structured physical activity, and psychosocial as well as family-based support, it is possible to optimize body composition and enhance bone metabolism. These comprehensive strategies not only contribute to improved bone mass in the short term but also play a vital role in maximizing PBM during adolescence and reducing the risk of osteoporosis later in life.

## 7 Summary

In summary, SO—a novel risk phenotype characterized by the synergistic interaction of “high fat and low muscle”—exerts adverse effects on bone health that are not merely additive. Instead, these effects are amplified through three interrelated mechanisms: insufficient mechanical loading, endocrine imbalance, and chronic inflammation, ultimately leading to impaired bone mass accumulation and reduced bone strength. Particularly during adolescence—a critical window for PBM formation—SO may have lasting effects on lifelong bone health. The core challenge in this field currently lies in the lack of unified, child-specific diagnostic criteria, which limits early identification and clinical intervention. From a clinical perspective, high-risk children should undergo routine assessment of muscle function, body composition, and bone health to enable early screening and risk stratification. Comprehensive management strategies centered on nutritional optimization, resistance and weight-bearing exercise training, and behavioral interventions involving family participation are expected to improve body composition and promote bone mass accumulation. Overall, deepening our understanding of SO in children will not only help optimize risk stratification but also provide a theoretical foundation for developing targeted intervention strategies, thereby promoting skeletal development and reducing the risk of lifelong bone fragility.

## 8 Limitations

Several limitations of the current literature on pediatric sarcopenic obesity (SO) should be acknowledged. First, the majority of studies are cross-sectional, which limits causal inference regarding the relationship between SO and skeletal outcomes. Second, there is a lack of standardized pediatric-specific definitions for SO, resulting in heterogeneity across studies. Third, measurement methods for muscle mass, strength, and bone outcomes vary widely, including DXA, MRI, BIA, and functional performance tests, further comparability. Fourth, longitudinal data on fracture risk and long-term skeletal outcomes are limited. Finally, confounding effects of puberty, hormonal changes, sex, and ethnicity are not consistently accounted for, which may influence observed associations. These limitations highlight the need for longitudinal, standardized studies that carefully adjust for developmental and hormonal factors to better inform clinical practice and intervention strategies.
