# Supplementary figures and images for: Sarcopenic obesity and skeletal development in children: recent advances
Source: Front Pediatr. 2026 Jul 13;14:1836925. doi: 10.3389/fped.2026.1836925 (PMC13402553; doi:10.3389/fped.2026.1836925)

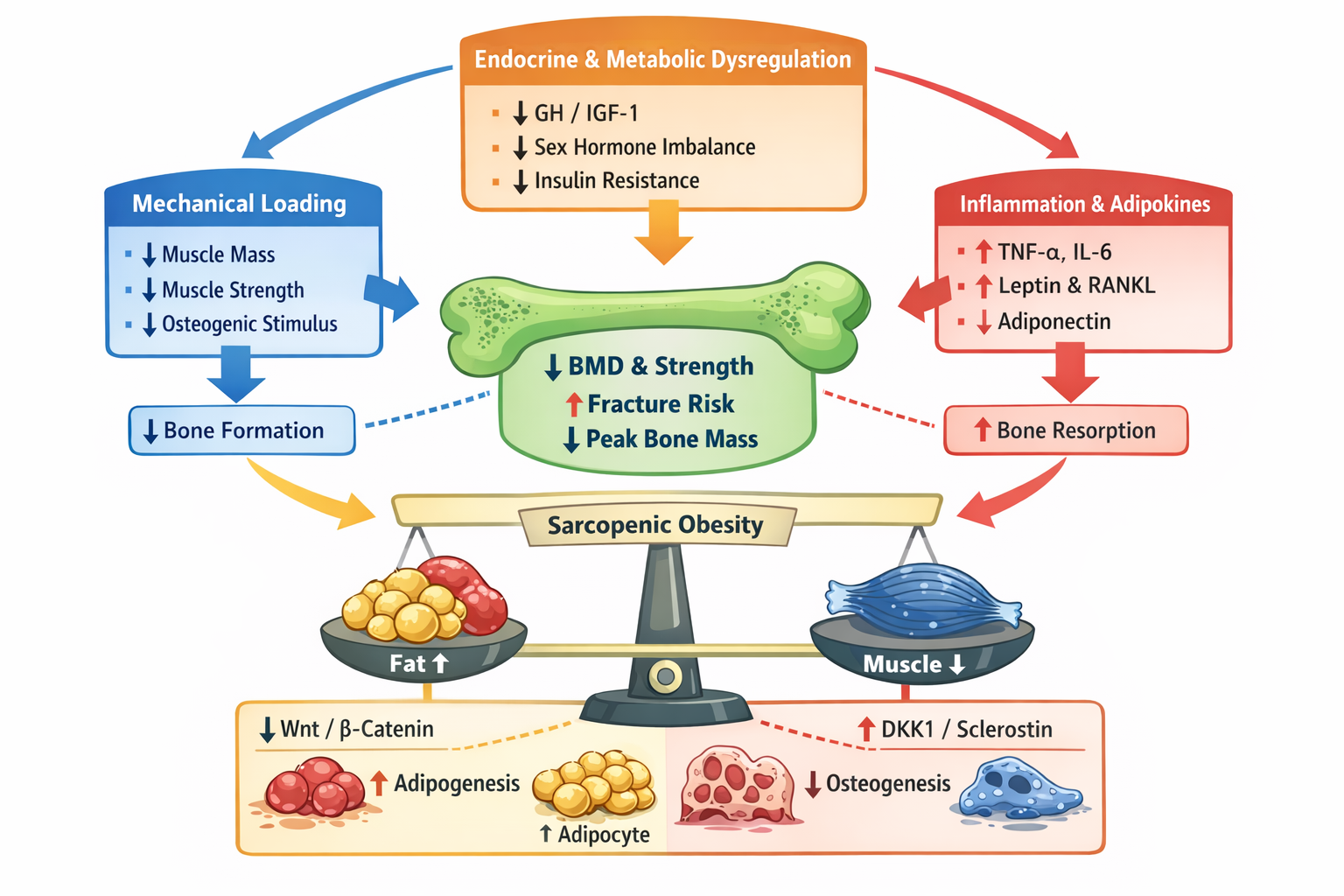

Supplement: Supplementary file 1 [file Image1.png]

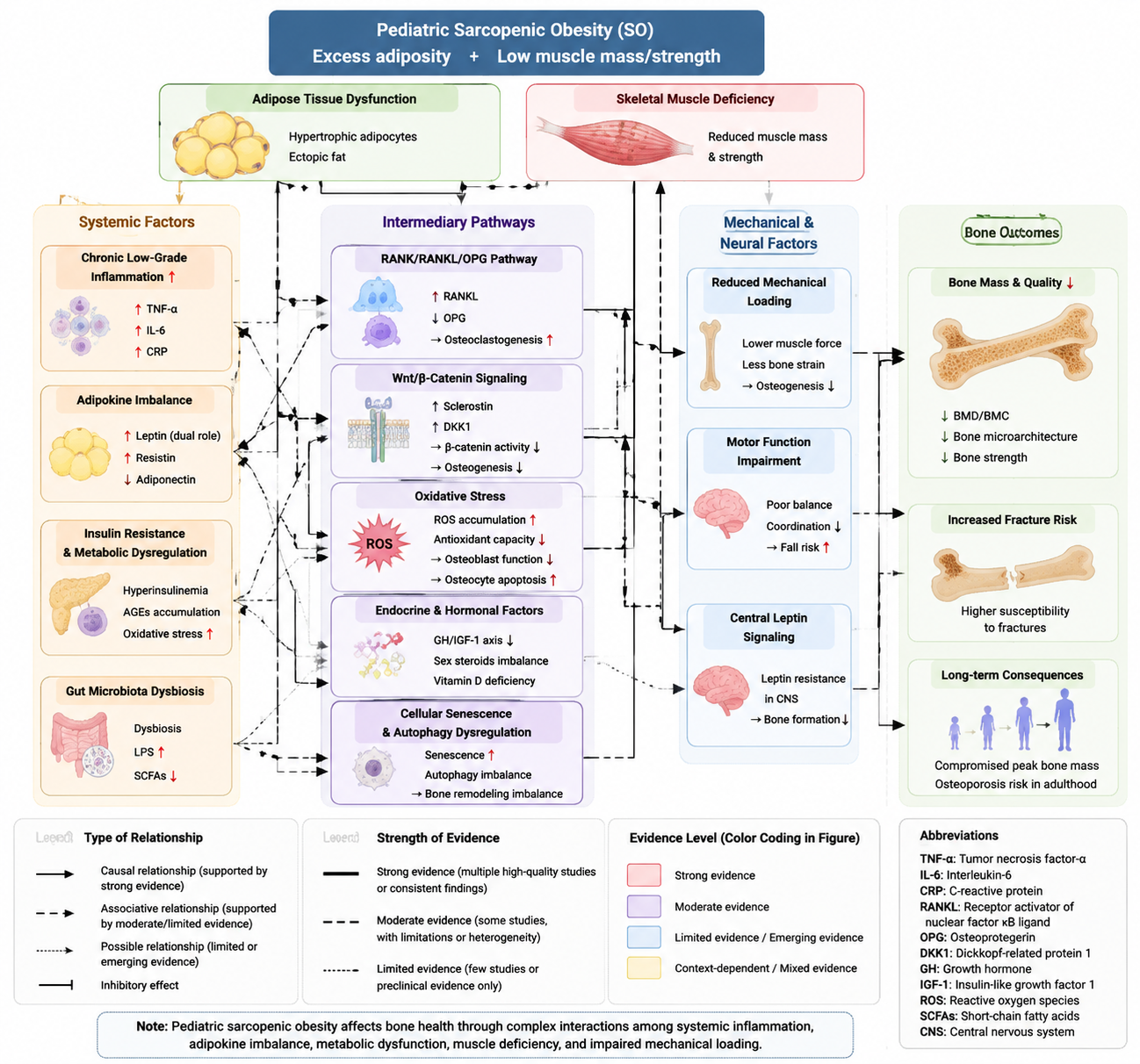

Supplement: Supplementary file 2 [file Image2.png]
